
JOURNAL INFORMATION
==============================
NLM Title Abbreviation: ScientificWorldJournal
Journal ID: ScientificWorldJournal
NLM Journal ID: 101131163
Journal ID: TSWJ

The Scientific World Journal

ISSN: 2356-6140
EISSN: 1537-744X
Publisher: Wiley

ARTICLE INFORMATION
==============================
PMCID: PMC5974863
PMID: 14755107
DOI: 10.1100/tsw.2003.113
Article ID: 184915
Article version: 1
Subjects: Editorial

Five Theories of the Human Existence

Ventegodt Søren 1 *
Andersen Niels Jørgen 2
Merrick Joav 3
1 The Quality of Life Research Center, Teglgårdstræde 4-8, DK-1452 Copenhagen K, Denmark
2 Norwegian School of Management, Sandvika, Norway
3 National Institute of Child Health and Human Development, Office of the Medical Director, Division for Mental Retardation, Ministry of Social Affairs, Jerusalem and Zusman Child Development Center, Division of Pediatrics and Community Health, Ben Gurion University, Beer-Sheva, Israel
*Søren Ventegodt: ventegodt@livskvalitet.org
Publication date: 2003
Electronic publication date: 2003 Dec 11
Volume: 3
First page: 1272
Last page: 1276
Copyright: Copyright © 2003 Soren Ventegodt et al.
Copyright year: 2003
License: This is an open access article distributed under the Creative Commons Attribution License, which permits unrestricted use, distribution, and reproduction in any medium, provided the original work is properly cited.
License URL: https://creativecommons.org/licenses/by/3.0/

Keywords: quality of life, QOL, philosophy, human development, holistic medicine, public health, Denmark

Funding: The 1991 Pharmacy Foundation Goodwill-Fonden JL-Foundation E. Danielsen and Wife’s Foundation Frimodt-Heineken Foundation Hede Nielsen Family Foundation Copenhagen Scientific Ethical Committee (KF)V.100.2123/91

==============================
Editorial
*TheScientificWorldJOURNAL* (2003) 3, 1272–1276
ISSN 1537-744X; DOI 10.1100/tsw.2003.113

### Five Theories of the Human Existence

Søren Ventegodt[1,*], Niels Jørgen Andersen[2], and Joav Merrick[3]
*[1]The Quality of Life Research Center, Teglgårdstræde 4-8, DK-1452 Copenhagen K, Denmark; [2]Norwegian School of Management, Sandvika, Norway; [3]National Institute of Child Health and Human Development, Office of the Medical Director, Division for Mental Retardation, Ministry of Social Affairs, Jerusalem and Zusman Child Development Center, Division of Pediatrics and Community Health, Ben Gurion University, Beer-Sheva, Israel*

**E-mail**: ventegodt@livskvalitet.org

*Received November 19, 2003; Accepted November 20, 2003; Published December 11, 2003*

**KEYWORDS:** quality of life, QOL, philosophy, human development, holistic medicine, public health, Denmark

**DOMAINS:** child health and human development, medical care, behavioral psychology, clinical psychology, nursing

### INTRODUCTION

It is not very often that new scientific medical theories about the human existence appear, but a series of small theoretical papers elaborating on the meaning of life[1] is now to be presented in this journal. The theories support another new theory that we have called the holistic process theory of healing[2,3]. Before you can understand the holistic process of healing or make it work in the clinic, you need to understand human existence thoroughly. You also need to understand it well enough to be able to explain it to your patients, together with the central concepts related to quality of life (QOL). We have developed the new theories of existence to obtain this clinical proficiency, so we have been forced to make them very clear and simple.

The first theories of existence of this kind were put forward by Carl Gustav Jung (1875–1961)[4], the famous student of Sigmund Freud (1856–1939). His well-known model of the structure of the ego and the true self are shown in Fig. 1[4]. We have now managed to make surprisingly simple models of the purpose of life[1]; the ego; the conditions for the unfolding of the unique talent of every human being; the problems of human development, especially in childhood; and finally, the nature of the evil side of man (the shadow, the anti-self) that collides with our deepest intention as human beings, i.e., trying to be and do good.

The theories of existence put forward are the results of a decade of research and philosophy in connection with quality of life, disease, and health[5,6,7,8,9,10,11], and clinical work with several hundreds of patients in holistic therapy using the models[12,13]. The intent that brought us to the theories was the intent of giving support, holding, and holistic medical treatment to our patients. Recently, the fruit of this positive development in Denmark, Norway, and Israel was expressed in the holistic process theory of healing[2]. This theory is now making the basis for the development of a clinical and holistic medicine[14,15,16,17].

*Corresponding author.
©2003 with author.

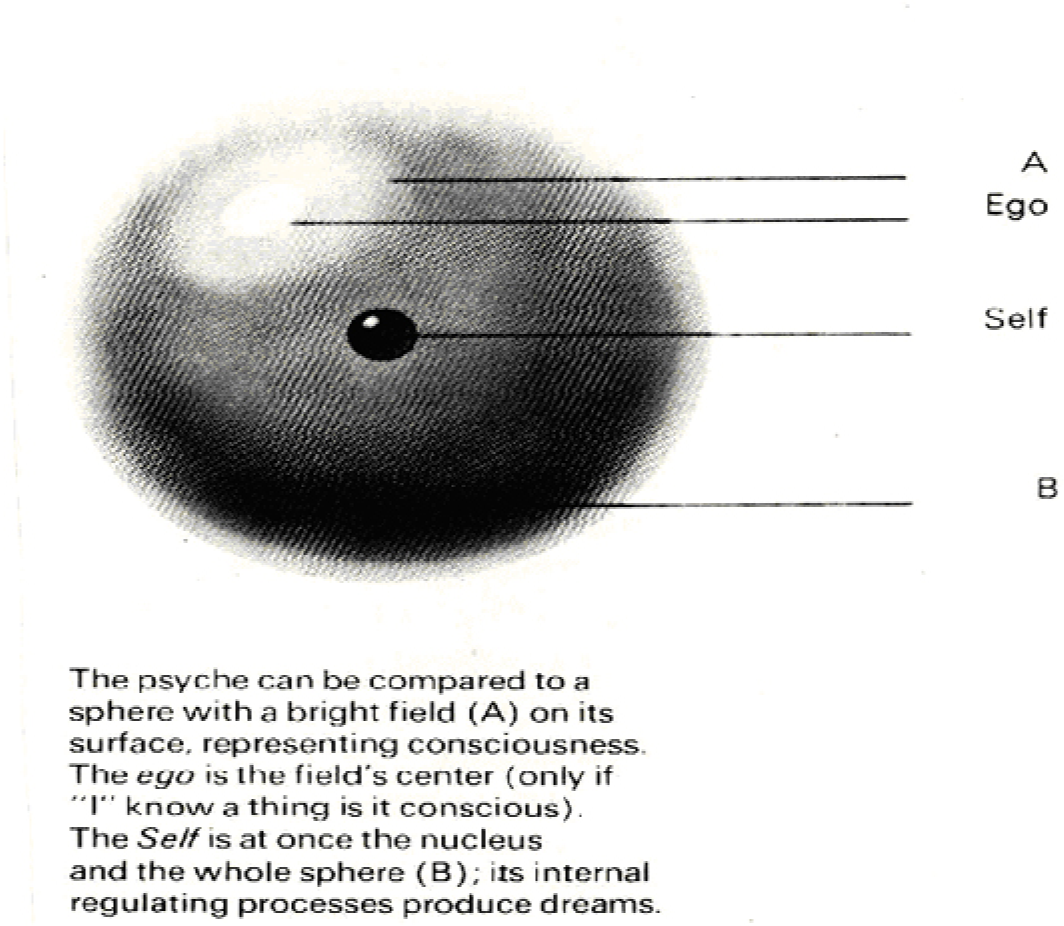

**FIGURE 1**. The famous, classical picture by C.G. Jung[4] of the vast human existence with the true self in the middle and the tiny ego on the surface (A), surrounded by a minute, white spot of consciousness, and besides that darkness and unconsciousness (B). The core of existence (the self) is similar to the purpose of life in the life mission theory[1].

The main argument why these five theories are important is that they seem to complement the reductionistic medical model of mainstream medicine in a beautiful and noncontradictory way. You are an organism containing advanced, genetically determined, biochemical pathways, and you are a living whole, conscious and alive, with love, feeling, and sexuality not reducible to mere chemistry.

Given the wide range of existential theories, some justification for choosing these five theories should be given, but this is difficult. Any theory that is not in conflict with known facts and that really helps the patients seems to be valid, and many such theories might be valid at the same time, as they express different perspectives on life and existence. So the argument here is not that we are now to present the final theories on human existence, but that we find these theories in accordance with known facts of life, and also that they are highly practical in our clinical work.

They seem to support a pretty theory of the holistic process of healing, which we have found to be of tremendous value to our chronic patients. This does not make the five theories true, but — in our opinion — gives a certain beauty to the five theories that is appealing. The abstract nature of the theories is also appealing because as soon as you understand the theories, many phenomena, which seemed complex and complicated before, now look simple and easy to understand. This is the case not only to us as therapists or consultants, but also to our patients and clients. This is the main reason why we find the five theories of existence useful and trustworthy.

### UNDERSTANDING HUMAN EXISTENCE

With this series of existential theories that we are going to present in this journal, we hope to show that human existence can be understood in depths. We also hope that this understanding of the human being, even in the most dark and hidden corners, will be joyful in the end. According to the life mission theory[1] and the derived theories, the human being is "good" and we believe that we all have the possibility for making true and ethical choices in life in order to get to know ourselves better, to integrate our hidden resources and talents (and also our black shadow) so that we can create the good life. The good life — where we become valuable for each other and to ourselves.

The theories are based on the life mission theory[1], which claims that from the beginning of his/her existence, the human being had a constructive purpose of life, which at the same time also is our greatest talent. When we learn to use this talent, we become not only happy, but also healthy and well functioning in our relations with ourselves and the surrounding world. When a human being denies and suppresses him- or herself, he or she becomes ill, unhappy, and malfunctioning. We believe that you need to find your true self and live in balance according with your life purpose, which will be the very best medicine[18,19] for you, even at the end of your life[20].

### TO REALIZE YOUR LIFE PURPOSE

However, it is not that easy to realize your life purpose because in our personal history, every human being has — at one point in time or another — suffered defeat or failed to realize the life purpose. According to the theory presented in this journal, an overwhelming pain early in life has caused a denial of the purpose. This denial is a highly self-destructive intention, which precisely balances against the constructive and more fundamental life purpose.

We sometimes find ourselves in an existential dilemma, where we cannot let go of the painful and often troublesome life purpose, because if we do that we will die, as the purpose of life is the only reason we have to live. The intended self-destruction is therefore the only way that the human being (in this case the child during childhood) can get out of the dilemma in order to survive and adapt. In defining a new life purpose, the earlier life purposes will be repressed, both the positive and the negative ones. The human being ends up being an adult unconscious about his or her strengths and weaknesses, about his or her "white and black" sides. This is exactly the scenario put forward by Jung (see Fig. 1).

### HOLISTIC PROCESS

In order to start all over again, the human being has to heal. The holistic process theory[2] explains how healing happens. When the human being gets and accepts the acknowledgment or respect and care that was originally lacking, these frozen memories from the past come to the surface of the consciousness and can now be integrated. From now on, the human being can return to his or her natural condition.

The holistic process of healing can be very effective in a group process and the last article explains which principles count in the existential group therapy. Principally, this therapy can be used on every human being, no matter which illness or sickness the human being suffers from.

This means we can offer an effective therapy, even on occasions where the etiology of the suffering is not understood. This means that the initial exploration by the medical diagnosticians, which is often a period of waiting for the patient, can happen at the same time of the treatment and therefore avoid a lot of expensive examinations, testing, and long hospitalization.

We have published two articles that document success in pilot studies with existential group therapy done with patients suffering from chronic pain and alcoholism[15,16]. Not only did the patients get healthier, but their quality of life improved. It looks as if holistic medicine can mobilize resources within the person.

### OUR HOPE

We hope the holistic medicine built on the new and deeper understanding of man will develop into an attractive complement and alternative to established biomedicine, which we would call the “new medicine”. In the Research Clinic for Holistic Medicine in Copenhagen, we are now developing new cures based on the holistic process theory for patients that cannot be helped sufficiently with biomedical drugs.

We want to help our patients to develop a high quality of life, better health, and an improved ability to function through the mobilization of the hidden resources within the patient, even in the last time of our lives[20].

We are going to present the following papers in this series as a continuation of the first paper on the life mission theory[1]:

- Ventegodt, S., Andersen, N.J., and Merrick, J. The Life Mission Theory II. The Structure of the Life Purpose and the Ego.
- Ventegodt, S., Andersen, N.J., and Merrick, J. The Life Mission Theory III. Theory of Talent.
- Ventegodt, S. and Merrick, J. The Life Mission Theory IV. Theory on Child Development.
- Ventegodt, S., Andersen, N.J., and Merrick, J. The Life Mission Theory V. Theory of the Anti-Self (the Shadow) or the Evil Side of Man.

### ACKNOWLEDGMENTS

Theses studies was supported by grants from The 1991 Pharmacy Foundation, as well as by supplementary grants from Goodwill-fonden, the JL-Foundation, E. Danielsen and Wife's Foundation, Emmerick Meyer's Trust, the Frimodt-Heineken Foundation, the Hede Nielsen Family Foundation, Petrus Andersens Fond, Wholesaler C.P. Frederiksens Study Trust, Else & Mogens Wedell-Wedellsborg's Foundation and IMK Almene Fond. Our research in quality of life has been approved by the Copenhagen Scientific Ethical Committee under number (KF)V.100.2123/91.

### REFERENCES

1. Ventegodt, S. (2003) The life mission theory. A theory for a consciousness-based medicine. *Int. J. Adolesc. Med. Health* **15,** 89–91.
2. Ventegodt, S., Andersen, N.J., and Merrick J. (2003) Holistic medicine III. The holistic process theory of healing. *TheScientificWorldJOURNAL* **3**, 1138-1146.
3. Ventegodt, S., Andersen, N.J., and Merrick, J. (2003) Holistic medicine IV. The principles of the holistic process of healing in a group setting. Accepted by *TheScientificWorldJOURNAL.*
4. Jung, C.G. (1964) *Man and His Symbols*. Anchor Press, New York.
5. Ventegodt, S., Andersen, N.J., and Merrick, J. (2003) Quality of life philosophy: when life sparkles or can we make wisdom a science? *TheScientificWorldJOURNAL* **3,** 1160-1163.
6. Ventegodt, S., Andersen, N.J., and Merrick, J. (2003) Quality of life philosophy I. Quality of life, happiness, and meaning in life. *TheScientificWorldJOURNAL* **3**, 1164-1175.
7. Ventegodt, S., Andersen, N.J., Kromann, M., and Merrick, J. (2003) Quality of life philosophy II. What is a human being? *TheScientificWorldJOURNAL* **3**, 1176–1185.
8. Ventegodt, S., Andersen, N.J., and Merrick, J. (2003) Quality of life philosophy III. Towards a new biology: understanding the biological connection between quality of life, disease, and healing. *TheScientificWorldJOURNAL* **3**, 1186–1198.
9. Ventegodt, S., Andersen, N.J., and Merrick, J. (2003) Quality of life philosophy IV. The brain and consciousness. *TheScientificWorldJOURNAL* **3**, 1199–1209.
10. Ventegodt, S., Andersen, N.J., and Merrick, J. (2003) Quality of life philosophy V. Seizing the meaning of life and becoming well again. *TheScientificWorldJOURNAL* **3**, 1210–1229.

11. Ventegodt, S., Andersen, N.J., and Merrick, J. (2003) Quality of life philosophy VI. The brain and consciousness. *TheScientificWorldJOURNAL* **3**, 1230–1240.
12. Ventegodt, S. (1999) *Philosophy of Life that Heals [Livsfilosofi der helbreder]*. Forskningscentrets Forlag, Copenhagen. [Danish]
13. Ventegodt, S. (2001) *Quality of Life as Medicine [Livskvalitet som medicin]* Forskningscentrets Forlag, Copenhagen. [Danish]
14. Ventegodt, S., Andersen, N.J., and Merrick, J. (2003) Holistic medicine: scientific challenges. *TheScientificWorldJOURNAL* **3**, 1108–1116.
15. Ventegodt, S., Merrick, J., and Andersen, N.J. (2003) Quality of life as medicine. A pilot study of patients with chronic illness and pain. *TheScientificWorldJOURNAL* **3**, 520–532.
16. Ventegodt, S., Merrick, J., and Andersen, N.J. (2003) Quality of life as medicine II. A pilot study of a five day “quality of life and health” cure for patients with alcoholism. *TheScientificWorldJOURNAL* **3,** 842–852.
17. Ventegodt, S., Clausen, B., Langhorn, M., Kromann, M., Andersen, N.J., and Merrick, J. (2003) Quality of life as medicine III. A qualitative analysis of the effect of a five days intervention with existential holistic group therapy: a quality of life course as a modern rite of passage. Submitted to *TheScientificWorldJOURNAL*.
18. Spiegel, D., Bloom, J.R., Kraemer, H.C., and Gottheil, E. (1989) Effect of psychosocial treatment on survival of patients with metastatic breast cancer. *Lancet* **2(8668),** 888–891.
19. Ornish, D. (1999) *Love and Survival. The Scientific Basis for the Healing Power of Intimacy.* HarperCollins, New York.
20. Merrick, J. and Ventegodt, S. (2003) What is a good death? To use death as a mirror and find the quality of life. *BMJ* October 31. On-line at:http://bmj.bmjjournals.com/cgi/content/full/327/7406/66

---

**This article should be referenced as follows**:

Ventegodt, S., Anderson, N.J., and Merrick, J. (2003) Five theories of the human existence. *TheScientificWorldJOURNAL* **3**, 1272–1276.

---

### BIOSKETCHES

**Søren Ventegodt, MD,** is the Director of the Quality of Life Research Center in Copenhagen, Denmark. He is also responsible for a Research Clinic for Holistic Medicine in Copenhagen and is a popular speaker throughout Scandinavia. He has published numerous scientific or popular articles and a number of books on holistic medicine, quality of life, and quality of working life. His most important scientific contributions are the comprehensive SEQOL questionnaire, the very short QoL5 questionnaire, the integrated QOL theory, the holistic process theory, the life mission theory, and the Danish Quality of Life Research Survey, 1991–94 in cooperation with the University Hospital of Copenhagen and the late pediatric professor Bengt Zachau-Christiansen. E-mail: ventegodt@livskvalitet.org. Website: www.livskvalitet.org/

**Niels Jørgen Andersen, MSc,** Professor, Department of Innovation and Economic Organization, Norwegian School of Management. This department conducts research and provides teaching in central topics related to innovation, business development, management of global companies, business history, and economic organization. Research activities within the Department are related to four core subjects within the discipline: business history, cooperative organizations, business development and entrepreneurship, and finally studies of industries with a special focus on the electricity industry. He is also the dynamic chairman of the nonprofit organization Stiftelsen Holistisk Medisin Scandinavia, which aims to support the scientific development, research, and documentation of complementary and holistic medicine in Scandinavia. E-mail: niels.j.andersen@bi.no. Website: www.bi.no/users/fgl93013/

**Joav Merrick, MD, DMSc,** is Professor of Child Health and Human Development affiliated with the Zusman Child Development Center, Division of Pediatrics and Community Health at the Ben Gurion University, Beer-Sheva, Israel; the Medical Director of the Division for Mental Retardation, Ministry of Social Affairs, Jerusalem; and the Founder and Director of the National Institute of Child Health and Human Development. He has numerous publications in the field of child and human development, rehabilitation, intellectual disability, disability, health, welfare, abuse, advocacy and prevention. Dr. Merrick received the Peter Sabroe Child Award for outstanding work on behalf of Danish Children in 1985 and the International LEGO-Prize (“The Children’s Nobel Prize”) for an extraordinary contribution towards improvement in child welfare and well being in 1987. E-mail: jmerrick@internet-zahav.net. Website: www.nichd-israel.com
